# Calendula Dressing

**Published:** 1899-08

**Authors:** Chas. B. Morrell

**Affiliations:** Hyde Park, Ohio


### CALENDULA DRESSING.

CHAS. B. MORRELL, M. D., HYDE PARK, OHIO.

Robert P——, aged 54, stepped out of a second-story stable door and landed on his heel. He suffered a compound dislocation of the ankle. The tibia was forced out of a ragged wound and completely denuded. Fibula fractured.

The man was hurt at three o'clock in the morning, nine miles from his home. He was carried that distance in a carriage with no other dressing than a piece of old cloth that was very dirty. The hemorrhage was profuse. I saw the man at eight o'clock. He was very weak, suffering from shock and loss of blood. The head of the tibia protruded from the wound nearly four inches. Hemorrhage on slightest touch. I adjusted a tourniquet bandage, dressed the wound with cotton dressing, and sent for assistance, deeming an amputation the only safe procedure. Counsel (Dr. C. A. Pauly) arrived at ten. After consultation it was decided that an amputation was not to be considered and the dislocation was reduced and the limb placed in a fracture box. As might be expected, suppuration set in with great vigor. The limb swelled, became œdematous and blotched, and with other symptoms, death seemed the only outcome of the case.

I determined to save the case, if possible, and chose as my principal remedies the Peroxide of Hydrogen and Calendula. The suppuration was rapid, involving the seat of the injury and extending up the limb following the track of the arteries. For nine days it seemed that the fight was lost, but my faith in these remedies, with indicated homœopathic remedies, which I will mention, never wavered, and on the tenth day I began to get results, and on the one hundred and first day the man walked out with a very good foot.

A slight deformity and a small open ulcer was all that was left to show the terrible ordeal, if I except the scars of the openings.

The treatment was simple. The Peroxide until the wound was perfectly clean whether it took an *ounce* or a *pound.* I used eleven pound packages.

After cleansing, I washed the cavities with an infusion of calendula and packed them with Halsey's surgical dressing. I could see the improvement every day. I believe that the *dressing* saved the case. The wound healed perfectly, even the openings throwing up healthy granulations and only in the seat of the injury were there any unhealthy granulations.

Internally I gave Arsenicum 3x, Baptisa 1x, China-off. 2x, and Hepar-sulph. 3x, winding up the case with Silicea 6x.—*The Medical Visitor.*

